# Heparin-Related Thrombocytopenia Triggered by Severe Status of Systemic Lupus Erythematosus and Bacterial Infection

**DOI:** 10.1155/2016/6571621

**Published:** 2016-09-06

**Authors:** Satoshi Suzuki, Shihoko Nakajima, Taiki Ando, Keisuke Oda, Manabu Sugita, Kunimi Maeda, Yutaka Nakiri, Yoshinari Takasaki

**Affiliations:** ^1^Department of Internal Medicine and Rheumatology, Juntendo University Nerima Hospital, Tokyo 177-8521, Japan; ^2^Department of Internal Medicine Research, Sasaki Institute, Sasaki Foundation, Tokyo 101-0062, Japan; ^3^Department of Internal Medicine and Rheumatology, Juntendo University School of Medicine, Tokyo 113-8431, Japan; ^4^Department of Emergency and Critical Care Medicine, Juntendo University Nerima Hospital, Tokyo 177-8521, Japan; ^5^Department of Internal Medicine and Nephrology, Juntendo University Nerima Hospital, Tokyo 177-8521, Japan

## Abstract

A patient with severe lupus nephritis developed thrombocytopenia during treatment with high-dose steroids. In addition to viral- or disease-induced cytopenia, the pathology was believed to arise from diverse contributing factors, such as thrombotic microangiopathy and heparin-related thrombocytopenia (HIT). By combining plasma exchange therapy and intravenous cyclophosphamide, we successfully controlled the SLE activity and improved the thrombocytopenia. An antecedent bacterial infection or SLE activity is believed to have contributed to the concurrent HIT.

## 1. Introduction

Systemic lupus erythematosus (SLE) is an autoimmune disease that can involve all bodily organs, including the lungs, kidneys, and skin. Diagnosis of SLE in Japan is primarily based on the diagnostic criteria of the American College of Rheumatology (ACR) as revised in 1997 [1]. However, there are cases that do not satisfy the diagnostic criteria yet involve symptoms or autoantibodies typical of SLE. Conversely, there are other autoimmune diseases, such as Sjögren's syndrome, that present with a pathology that would end up being misdiagnosed as SLE when judged purely on the diagnostic criteria [2]. Recently, the Systemic Lupus Collaborating Clinics (SLICC) have proposed new criteria for classifying SLE [3], but neither their sensitivity nor their specificity greatly exceeds those of the ACR diagnostic criteria. There are also individual differences in how symptoms manifest, varying from mild pathology limited to skin rashes and arthritis to severe pathology involving major organs. Accordingly, treatment needs to be tailored to suit individual symptoms. SLE is a refractory autoimmune disease that challenges a rheumatologist's competence in both diagnosis and treatment.

Representative forms of severe pathology with organ involvement include central nervous system lupus and glomerulonephritis (lupus nephritis), but patients so often exhibit severe thrombocytopenia that has also been included in the diagnostic criteria.

## 2. Case Report

**Figure 1:**
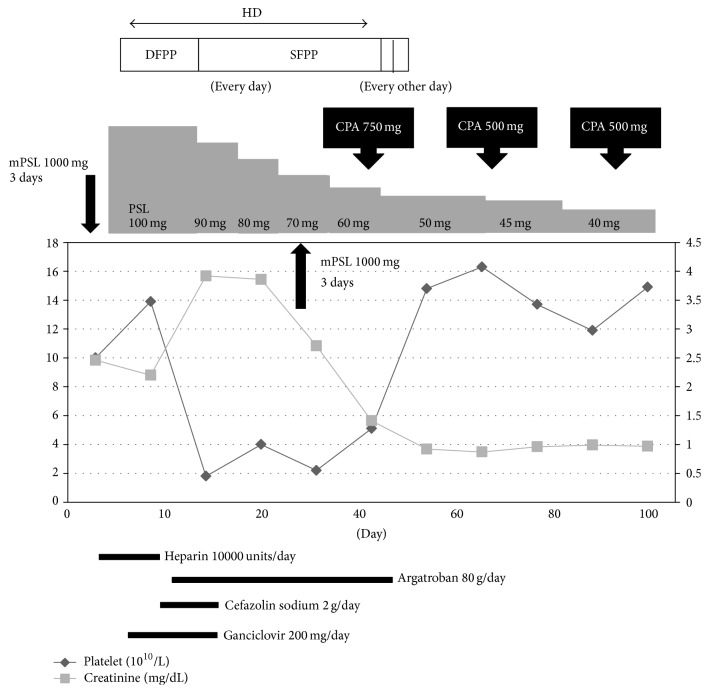
Clinical course.

A 52-year-old man with an unremarkable past medical history had anemia noted in a 2014 health check and, by the end of the year, lower limb edema was observed but was left untreated. In March 2015, he was admitted to a nearby clinic with a diagnosis of nephrotic syndrome. Renal dysfunction was observed when the nephrotic syndrome was diagnosed, and blood purification therapy was being considered, for which he was transferred to our hospital. Since progressive renal dysfunction had been observed, methylprednisolone (mPSL) pulse therapy (mPSL 1000 mg/day for 3 days) was initiated on the day of hospital transfer. This was followed by treatment with 100 mg/day (1 mg/kg) of prednisolone (PSL), beginning on hospital day 4. Testing conducted in parallel showed anti-nuclear antibody titers of ×320 (Homo ×320, Spe ×320), anti-ds DNA antibody (ELISA) 300 IU/mL, anti-cardiolipin antibodies (IgG-aCL) at 16 U/mL, leukocytes at 1000/L, platelets at 100,000/L, and urine protein levels of 2.6 g/gCre, and he was diagnosed with SLE. With such significant systemic edema, difficulty with hemostasis was anticipated, and the patient was unable to lie prone, making it impossible to obtain a renal biopsy to differentiate the type of lupus nephritis. High disease activity persisted, and plasma exchange therapy (double filtration plasmapheresis) was initiated on hospital day 9. On hospital day 11, dialysis (hemodialysis, HD) was initiated. A fever was observed on hospital day 21, and* Staphylococcus aureus* (methicillin-sensitive* Staphylococcus aureus*, MSSA) was detected from a blood culture. At first, vancomycin was selected as an antibiotic, but after sensitivity was confirmed, the antibiotics were deescalated to cefazolin. On hospital day 23, decreases in oxygen saturation and blood pressure were observed, and the patient was admitted to the intensive care unit (ICU) with congestive heart failure. His circulation was supported medically and the antibiotics were continued. The patient's testing did not support a diagnosis of acute coronary syndrome nor poor drainage during HD; myocardial damage from cytokine storm was believed to be the cause of his heart failure. High-level SLE activity persisted, and the patient was deemed to have a steroid-resistant pathology. We considered introducing cyclophosphamide (CPA) or mycophenolate mofetil (MMF). Laboratory test findings are shown in Table 1. Clinical course is shown in Figure 1.

Platelet count had been gradually decreasing since hospital day 10, and no improvement was observed despite changes in and discontinuation of the drugs used. A search for the cause of his thrombocytopenia identified CMV antigenemia (C7-HRP: 3/50,000 infected cells). He was also positive for CMV-DNA, and the possibility of thrombocytopenia associated with CMV viremia was considered. ADAMTS13 activity, which was submitted at the same time, also exhibited a mild decrease at 53%, and concurrent thrombotic microangiopathy (TMA) was considered, in light of the findings of fever, peripheral blood schistocytes, hemolytic anemia, confusion, and progressive renal failure. In addition, he was found to be positive for heparin-related thrombocytopenia (HIT) antibodies, and we considered the possibility that he was experiencing HIT caused by the heparin used in thrombosis prophylaxis since hospital day 2. We found a deep vein thrombosis at left popliteal artery by ultrasonography, so 4T's score ran to 7 points. The viremia forced cyclophosphamide and MMF to be postponed, and simple plasma exchange therapy (single filtration plasmapheresis, SFPP) was initiated on hospital day 23 while the patient also was undergoing antiviral therapy with ganciclovir. Beginning on hospital day 24, a second course of mPSL pulse therapy (mPSL 1000 mg/day for 3 days) was begun to treat the cytokine storm, following which the steroid dosage would be reduced. Vital signs stabilized, and he was discharged from the ICU on hospital day 29, but hemoptysis occurred on hospital day 31 while he was undergoing HD. He was found to have decreased oxygen saturation and a decreased level of consciousness and started on ventilator management with endotracheal intubation. Chest computed tomography and bronchoscopy identified an alveolar hemorrhage, and again he was admitted to the ICU. Although this hemorrhage was believed to be the result of his low platelet count, he still had high SLE activity, and the possibility of alveolar hemorrhage secondary to SLE was considered. On hospital day 36, CMV C7-HRP was still positive; we made the decision to perform intermittent intravenous therapy (intravenous cyclophosphamide therapy, IVCY) with 750 mg of CPA to control SLE activity. Later, platelet count increased, in tandem with improved TMA pathology due to SFPP, reduced HIT due to heparin cessation, and an improvement in viremia due to a relatively fast decrease in steroid dosage.

The patient had a favorable therapeutic response to cyclophosphamide, and we were able to conclude mechanical ventilation and discharge him from the ICU on hospital day 39. We also discontinued HD and SFPP, and he underwent a second course of IVCY on hospital day 71 and a third course on hospital day 107, with a favorable course that allowed him to be discharged from the hospital on hospital day 127. Medical treatment has been continuing on an outpatient basis; SLE has maintained low disease activity, and there has been no decrease in his platelet count.

## 3. Discussion

Cytopenias frequently complicate SLE, including anemia (63.0%), lymphopenia (40.3%), leukopenia (30.0%), and thrombocytopenia (10.9%) [4]. In particular, thrombocytopenia has very diverse causes, including hemophagocytic syndrome (HPS), TMA, and antiphospholipid antibody syndrome, all involving the underlying disease, and viral infection, drug-induced cytopenia, and thrombosis caused by steroids, which involve the treatment. Depending on the cause, steroids and other such immunosuppressive therapies may be effective, but they have an inverse effect if the cause is infection or thrombosis [5]. The present case is believed to have had simultaneous onset of TMA and HIT, in addition to viral infection. TMA is a severe manifestation of SLE pathology, and, if left untreated, it is frequently fatal. TMA occurs frequently in highly active SLE with renal complications [6]. SFPP is an effective treatment for TMA and lowers the mortality rate, said to be 85–100% in the absence of treatment, down to 10–30% [7–9]. However, there are cases where SFPP is ineffective at treating or stopping recurrence, and in such cases, rituximab is reportedly effective [10, 11]. HIT is a pathology where, for some reason, pathogenic HIT antibodies are produced out of the autoantibodies (anti-heparin/PF4 antibodies) against platelet factor 4 (PF4) and the heparin complex [12]. HIT antibodies activate the vascular endothelium and induce thrombosis from excessive thrombin production [12]. To diagnose HIT, it is useful to measure HIT antibodies directly, a test which is covered by insurance even in Japan, but it is necessary to have a comprehensive approach by combining the 4T's score using the extent of thrombocytopenia, history of heparin use, and the presence or absence of thrombosis [13]. Anticoagulation therapy is required for thrombosis prophylaxis, but heparin exacerbates the thrombocytopenia, in which case argatroban, which is a thrombin inhibitor, is used. Approximately 19% of SLE patients have antibodies against PF4 (anti-PF4 antibodies) [14]. Anti-PF4 antibodies are believed to be synonymous with anti-heparin/PF4 antibodies in that they react to the heparin/PF4 complex (are heparin-dependent). However, heparin-independent anti-PF4 antibodies, which are believed to react only to PF4, have been discovered recently. The appearance of heparin-dependent anti-PF4 antibodies is associated with thrombocytopenia, while the appearance of heparin-independent anti-PF4 antibodies is related to SLE disease activity [14]. The patient presented tested positive for HIT antibodies, and therefore it was believed that autoantibodies against the heparin/PF4 complex (heparin-dependent anti-PF4 antibodies) were being produced, even though disease activity remained extremely high. Anti-heparin/PF4 antibodies are reportedly produced due to a cross-reaction between the complex of PF4 and bacteria (in particular,* Staphylococcus aureus* and* Escherichia coli*) [15]. This patient developed MSSA bacteremia, and it was possible that he experienced an abnormal immune response to the complex of bacteria and PF4 and produced anti-heparin/PF4 antibodies due to enhanced SLE activity. The pathology presented with the production of diverse autoantibodies, including anti-DNA, IgG-aCL, and HIT antibodies, and he was believed to have an abnormal enhancement of B-cell function. A malignant lymphoma test (7-amino-actinomycin-D, 7AAD) performed on peripheral blood ruled out B-cell lymphoproliferative disease. Finally, he responded to cyclophosphamide, which is a DNA synthesis inhibitor; in such cases, we feel that a treatment strategy targeting B-cells, such as rituximab or belimumab, may be effective and useful in terms of the risk of adverse reactions.

## 4. Conclusion

The causes of thrombocytopenia complicating SLE with high disease activity include those associated with the underlying disease, but numerous reports show that TMA, HPS, and similar conditions are also possible. In the present case, HIT antibodies appeared, and the patient is believed to have had concurrent heparin-related thrombocytopenia. In patients who are receiving high-dose steroids and have a high risk of thrombosis, there are some cases where heparin is used prophylactically, but we feel it is necessary to pay attention to the onset of heparin-related thrombocytopenia when the patient is being treated for autoimmune disease with high disease activity or when relatively severe bacterial infection is concurrent.

## Figures and Tables

**Table 1:** Laboratory test findings.

Test	Result
Blood cell count	WBC 1000/*μ*L
	Neu 505/*μ*L
	Lym 100/*μ*L
	Eosi 0/*μ*L
	RBC 280万/*μ*L
	Hb 6.9 g/dL
	Hct 24.3%
	Plt 10000/*μ*L

Coagulation	PT 114%
	(PT-INR 0.95)
	APTT 30.5 sec
	FDP 25.2 *μ*g/mL

Biochemistry	T-Bil 0.3 mg/dL
	D-Bil 0.1 mg/dL
	AST 22 IU/L
	ALT 11 IU/L
	*γ*-GTP 21 IU/L
	BUN 62 mg/dL
	Cre 2.46 mg/dL
	eGFR 23.31
	UA 10.4 mg/dL
	Na 134 mEq/L
	K 5.0 mEq/L
	Cl 105 mEq/L
	TP 5.0 g/dL
	Alb 1.9 g/dL

Serology	CRP 1.87 mg/dL
	ANA ×320
	Homogeneous ×320
	Speckled ×320
	Anti-DNA antibody 300 IU/mL
	Anti-RNP antibody (−)
	Anti-SS-A antibody (−)
	Anti-cardiolipin antibody (IgG) 16 IU/mL
	CH50 12/mL
	C3 24 mg/dL
	C4 5 mg/dL
	IgG 1554 mg/dL
	IgA 148 mg/dL
	IgM 290 mg/dL

Urinalysis	pH 5.0
	Protein (4+)
	Ketone body (−)
	Occult blood (±)

Urinary sediment	RBC 10–19 HPF
	WBC 20–29 HPF
	Hyaline cast (2+)
	Epithelial cast (1+)
	Granular cast (1+)
	Waxy cast (1+)

WBC: white blood cell, RBC: red blood cell, Hb: hemoglobin, Hct: hematocrit, Plt: platelet, PT: prothrombin time, INR: international normalized ratio, APTT: activated partial thromboplastin time, FDP: fibrinogen and fibrin degradation products, Bil: bilirubin, AST: aspartate transaminase, ALT: alanine transaminase, BUN: blood urea nitrogen, eGFR: estimated glomerular filtration rate, TP: total protein, CRP: C-reactive protein, ANA: anti-nuclear antibody, LAC: lupus anticoagulant, CH50: total complement activity, Ig: immunoglobulin, and HPF: high power field.
